# Detection, molecular function and mechanisms of m5C in cancer

**DOI:** 10.1002/ctm2.70239

**Published:** 2025-02-26

**Authors:** Linhui Zhang, Yuelong Li, Liqing Li, Fei Yao, Maoping Cai, Dingwei Ye, Yuanyuan Qu

**Affiliations:** ^1^ Department of Urology Fudan University Shanghai Cancer Center Shanghai China; ^2^ Department of Oncology Shanghai Medical College Fudan University Shanghai China; ^3^ Shanghai Genitourinary Cancer Institute Shanghai China; ^4^ Department of Nursing Fudan University Shanghai Cancer Center Shanghai China

**Keywords:** 5‐methylcytosine, biologic functions, cancer, detection techniques, RNA modification

## Abstract

Interest in RNA posttranscriptional modifications, particularly 5‐methylcytosine (m5C), has surged in recent years. Studies have shown that m5C plays a key role in cellular processes and is closely linked to tumourigenesis. This growing focus emphasises the importance of understanding the diverse impacts of m5C modifications in both normal cellular functions and cancer development. Moreover, strides in methodologies for discerning m5C have facilitated intricate transcriptome cartography of RNA methylation at the solitary nucleotide echelon. This technical progress has fueled a surge in m5C‐centric investigations, facilitating further exploration of this RNA modification. This review provides a comprehensive analysis of the oncogenic potential of m5C RNA modification, elucidating the precise molecular mechanisms driving its role in cancer development. It consolidates current knowledge regarding the biological consequences of m5C RNA modification in tumour cells. Understanding the role of methylation‐related processes in tumourigenesis shows promise for advancing cancer diagnosis and therapeutic strategies.

**Highlights:**

- m5C modifications are dynamically regulated by writers, readers, and erasers, influencing cancer progression, metastasis, and immune evasion.
- Distinct m5C regulatory networks exist across cancers, modulating oncogenic pathways and therapy responses.
- m5C signatures serve as biomarkers for cancer prognosis and treatment stratification, highlighting their role in precision oncology.

## Graphical abstract

**The role of m5C in cancers**. The role of m5C regulators‐ writers, erasers, and readers‐ in various cancer types, including NSCLC, PCa, GC, AML, BCa, and HCC.

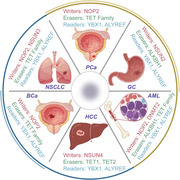

## 1 INTRODUCTION

RNA modifications represent a vital layer of gene expression regulation, shaping RNA stability, translation and interactions.^1^ Since pseudouridine was first identified in 1951 as the earliest known post‐transcriptional RNA modification,^2^ over 170 chemical modifications have been discovered across various RNA types, including messenger RNA (mRNA), transfer RNA (tRNA), ribosomal RNA (rRNA) and long non‐coding RNA (lncRNA).^3^ Among these, N6‐methyladenosine (m6A) and 5‐methylcytosine (m5C) have garnered particular attention for their dynamic and reversible nature, allowing precise regulation of RNA function. These modifications play critical roles in processes such as RNA stability, translation efficiency and splicing, underscoring their importance in maintaining cellular homeostasis and driving disease progression.^4^, ^5^

m5C, defined by the methylation of the fifth carbon in cytosine residues, plays a role in numerous physiological and pathological processes, including tumourigenesis. This modification occurs across different RNA species, and is mediated by specific ‘writer’ enzymes such as NSUN2 and DNMT2, ‘eraser’ enzymes such as TET and ‘reader’ proteins such as ALYREF.^6^, ^7^, ^8^, ^9^ Dysregulation of these components can significantly impact RNA metabolism and cellular processes, contributing to cancer initiation and progression.

Despite its emerging significance, the precise mechanisms and biological implications of m5C remain underexplored. Recent technological advancements, such as bisulphite sequencing and liquid chromatography‒tandem mass spectrometry (LC‒MS/MS), have enabled high‐resolution mapping of m5C modifications, providing novel insights into its functional roles.^10^, ^11^ However, many questions remain unanswered, particularly regarding the interplay between m5C and other RNA modifications and its contributions to oncogenic pathways.

This review provides a comprehensive overview of m5C RNA modification, delving into its specific molecular mechanisms and contributions to cancer progression. By synthesising current research, it highlights the biological effects of m5C in tumour cells, shedding light on its regulatory functions in key processes such as RNA stability, translation and gene expression. Additionally, the review emphasises the significance of m5C‐related methylation in tumourigenesis, offering valuable insights into its potential as a biomarker for cancer diagnosis and a target for therapeutic intervention. This expanded understanding aims to bridge knowledge gaps and inspire future studies to advance precision medicine in oncology.

## 2 RNA METHYLATION

RNA methylation stands as a meticulously scrutinised alteration in the realm of RNA modifications, with recent advancements in RNA methylation research due to the development of high‐throughput sequencing technology.^12^ It has been established that RNA methylation is associated with various human physiologies and diseases, particularly tumour immunity.^13^ The regulation of normal physiological processes relies on the important role of RNA methylation, while abnormal methylation can lead to diseases and cancers. RNA methylation can be categorised into various forms based on the locus of methylation, including N1‐methyladenosine (m1A), m5C, m6A, N7‐methylguanosine (m7G) and N6,2′‐O‐dimethyladenosine (m6Am). RNA modification proteins (RMPs) exert a pivotal influence in facilitating the dynamic and reversible methylation of RNA.^14^

**FIGURE 1:**
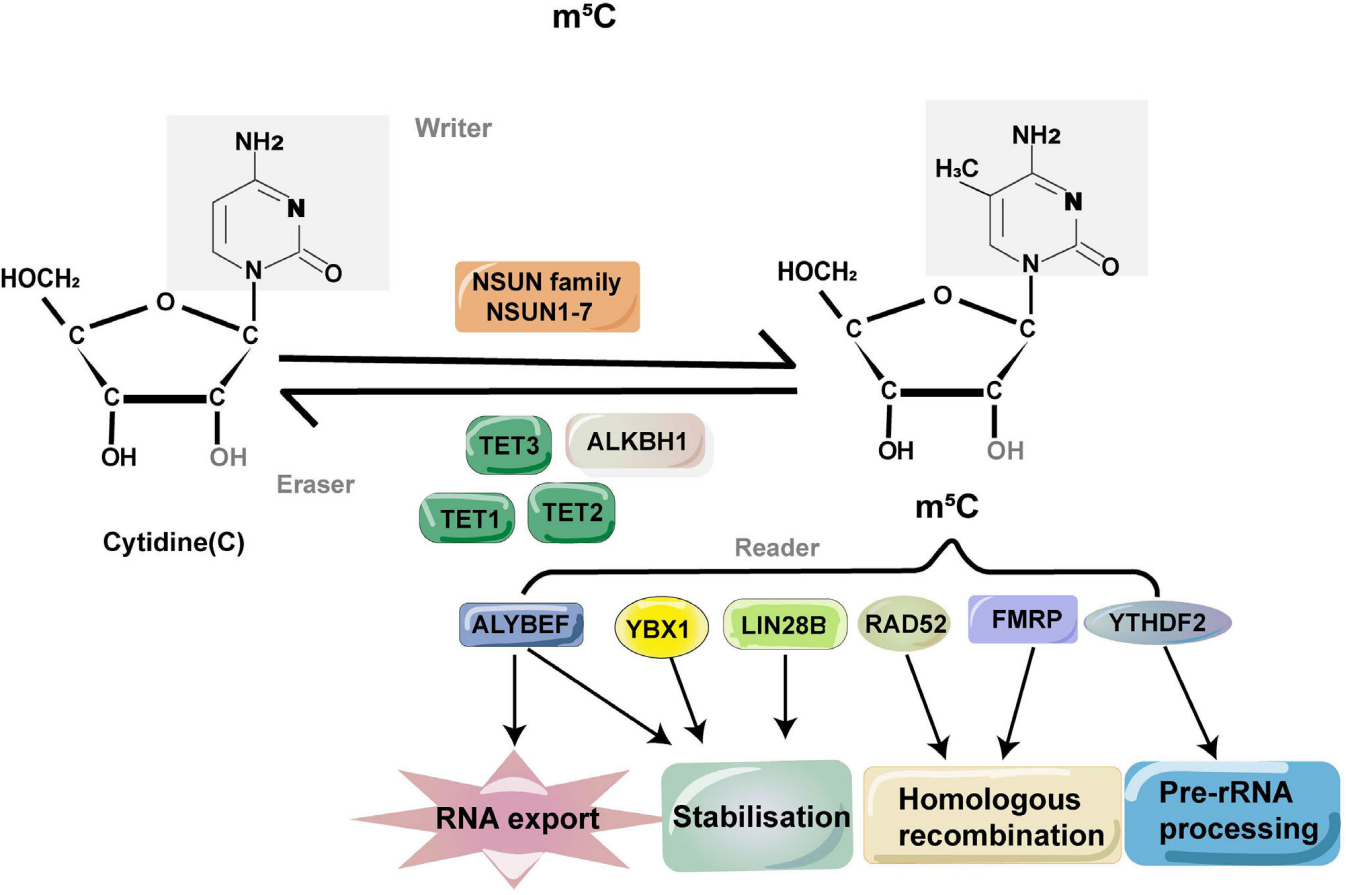
Overview of 5‐methylcytosine (m5C). Dynamic regulation of m5C RNA modification. The ‘writers’ (e.g., NSUN family, NSUN1‐7) catalyse the methylation of cytosine residues, while ‘erasers’ (e.g., TET1/2/3, ALKBH1) demethylate m5C, allowing for reversibility. ‘Readers’ (e.g., ALYREF, YBX1, LIN28B, RAD52, FMRP, YTHDF2) bind to m5C‐modified RNAs and mediate downstream processes. These include RNA export, transcript stabilisation, homologous recombination and pre‐rRNA processing. Dysregulation of these pathways contributes to various cancers through effects on gene expression, RNA metabolism and tumour microenvironment interactions.

Methylcytosine methylation, also known as m5C, is a common modification that occurs on the fifth carbon atom of cytosine. This modification is found in various types of RNAs, involving mRNA, tRNA, rRNA, viral RNA, vault RNA and lncRNA. These diverse RNAs play important roles in maintaining RNA constancy and augmenting translational efficacy.^15^ The induction of m5C methylation is orchestrated by methyltransferases, which are referred to as ‘writer’ enzymes. Examples of these enzymes include NSUN and members of the DNMT family. On the other hand, demethylases such as the TET families and binding proteins such as YBX1 function as ‘eraser’ and ‘reader’ proteins, respectively, dynamically regulating the m5C methylation process^16^ (Figure 1). The significance of m5C in modulating gene expression, metabolism and diseases has been extensively documented.^17^, ^18^ In the realm of oncology, the post‐transcriptional alteration of RNA has long been acknowledged for its pivotal roles in the progression and pathological mechanisms across diverse cancer types,^19^ offering innovative pathways for mechanistic exploration and therapeutic progress. Variations in m5C modifications within both coding and non‐coding RNA molecules exhibit intimate correlations with cellular proliferation, metabolic dynamics, tumour dissemination and are evident in various malignancies,^20^ including hepatocellular carcinoma (HCC),^21^ breast cancer (BC)^22^ and bladder cancer (BCa).^23^ Additionally, m5C exerts a substantial impact on a myriad of immune cell populations, encompassing B cells, T cells, NK cells, granulocytes, macrophages and others.^24^

## 3 DETECTION METHODS OF M5C

Delving into modified target genes is imperative for thoroughly probing the biological ramifications of mRNA m5C alterations, particularly in the field of cancer research. Recent advancements in sequencing technologies and experimental methodologies have enabled the qualitative and quantitative analysis of mRNA methylation at the individual nucleoside tier, furnishing unparalleled elucidations into its functional capacities.^25^ In recent times, various robust detection approaches are utilised to identify m5C modifications, including: (1) physicochemical techniques embracing chromatography, mass spectrometry (MS),^26^ high‐performance liquid chromatography (HPLC)^27^ and LC‒MS/MS^28^; (2) chemical conversion approaches merging next‐generation sequencing (NGS) methodologies such as RNA bisulphite sequencing (RNA‐BisSeq),^29^ ten‒eleven translocation (TET)‐assisted peroxotungstate oxidation sequencing (TAWO‐seq)^30^ and ultrafast bisulphite sequencing (UBS‐seq)^31^; (3) immunoprecipitation coupled with NGS, including 5‐aza‐immunoprecipitation (5‐Aza‐seq)^32^ in conjunction with m5C individual‐nucleotide‐resolution cross‐linking and immunoprecipitation (miCLIP)^33^; (4) third‐generation sequencing (TGS), leveraging differences in electrical signals, as seen in Nanopore‐seq^34^; (5) predictive models powered by machine learning, such as PEAm5C, m5C‐PseDNC (5‐methylcytosine site prediction using pseudo‐nucleotide compositions),^35^ Pm5CS‐Comp‐mRMR (minimum redundancy maximum relevance), identifying RNA m5C sites via pseudo‐nucleotide compositions (iRNA‐m5C‐PseDNC)^36^ and m5CHPCR (heuristic reduction of nucleotide physicochemical properties) (Table 1).^37^ These methodologies collectively constitute an extensive arsenal frequently utilised for the systematic identification of m5C modifications within designated genetic loci. This integrative approach allows for a nuanced understanding of the complex landscape of mRNA modifications and their functional consequences. Such insights are instrumental in advancing our knowledge of cancer‐related epigenetic alterations, offering novel breakthroughs and potential strategies for targeted therapies.

This section categorises these methodologies into distinct groups, starting from traditional physicochemical techniques to cutting‐edge sequencing methods and computational tools. By systematically examining their relevance to m5C detection, sample preparation requirements, mechanisms and practical applications, this section aims to provide a comprehensive framework for understanding the current capabilities and limitations of m5C detection technologies.

### 3.1 Physicochemical methods

Physicochemical methods are critical tools for identifying and characterising m5C modifications in RNA. These techniques are renowned for their high sensitivity, specificity and quantitative capabilities, making them ideal for the precise detection of RNA modifications.

#### 3.1.1 High‐performance liquid chromatography

HPLC is a highly sensitive analytical technique capable of detecting substances at nanogram levels. In this method, RNA samples are enzymatically digested into nucleosides using nucleases such as RNase T2 or nuclease P1. The resulting nucleosides are separated based on their hydrophobicity using a chromatographic column under optimised conditions, such as gradient elution with buffers of varying ionic strength. m5C is specifically detected by its unique retention time, which is compared against standard references to confirm its identity. Quantification is achieved by integrating the peak areas corresponding to m5C, often calibrated against synthetic standards. This technique has been extensively used for studying RNA modifications, particularly in small RNA species like tRNA and rRNA. For example, researchers used HPLC to determine the major and minor DNA base composition of 17 thermophilic bacteria.^38^

#### 3.1.2 Mass spectrometry

MS provides a direct and highly accurate method for detecting m5C modifications.^39^ RNA is first digested enzymatically into individual nucleosides, as purity is critical to minimise ion suppression during analysis. The nucleosides are ionised, typically using electrospray ionisation, and analysed in the mass spectrometer. m5C is identified by its distinct mass‐to‐charge ratio (m/z), with high‐resolution MS capable of distinguishing closely related methylated and unmethylated cytosines. Tandem MS (MS/MS) is employed to further fragment the molecule, confirming the modification by analysing its fragmentation pattern. This method is widely used due to its sensitivity and specificity, although it requires access to advanced instrumentation.

As a specialised form of MS, LC‒MS/MS integrates the separation efficiency of LC with the analytical precision of MS.^40^ This method uses enzymatic RNA digestion and stable isotope‐labelled nucleoside standards to enhance quantification accuracy. LC isolates m5C based on its physicochemical properties, effectively reducing background noise and enhancing the clarity of the MS signal. The MS/MS mode provides additional structural confirmation by analysing the fragmentation patterns of m5C.^41^

LC‒MS/MS is one of the most widely used MS techniques in epigenetic research due to its high sensitivity, strong resolution and broad applicability. It is extensively applied in studies involving DNA methylation, histone modifications and RNA modifications. This method offers both qualitative and quantitative insights, making it especially suitable for analysing complex RNA populations, such as transcriptome‐wide studies.^42^ For instance, LC‒MS/MS has been used to study the dynamic changes of m5C mRNA in different tissues and developmental stages of *Arabidopsis thaliana*.^28^

### 3.2 Chemical conversion methods

Chemical conversion methods are specialised techniques that rely on chemical reactions to selectively modify nucleotides, enabling the detection and quantification of m5C in RNA. These techniques exploit the unique reactivity of m5C compared to unmodified cytosines, enabling precise analysis.

#### 3.2.1 RNA bisulphite sequencing

RNA‐BisSeq is the gold standard for single‐base m5C detection. The method involves treating RNA with bisulphite, a reagent that deaminates unmethylated cytosines into uracil, while leaving m5C unmodified. Following conversion, the RNA is reverse transcribed into cDNA and sequenced. By comparing the sequence of treated and untreated RNA, m5C sites are identified. The efficiency of bisulphite conversion is critical, and incomplete conversion can lead to false positives. Computational tools are often employed to correct for such artefacts. RNA‐BisSeq has been used extensively in transcriptome‐wide studies. For example, it identified target genes involved in Epidermal Growth Factor Receptor Tyrosine Kinase Inhibitor (EGFR‐TKI) resistance by NSUN2 in EGFR‐mutant non‐small‐cell lung cancer.^43^

#### 3.2.2 TAWO‐seq

TAWO‐seq employs chemical oxidation to convert m5C into 5‐hydroxymethylcytosine (5‐hmC), which is then sequenced. RNA is treated with peroxotungstate, a specific oxidant, under conditions that preserve RNA integrity. Following oxidation, RNA is subjected to library preparation and high‐throughput sequencing. The chemical specificity of the oxidation step ensures accurate identification of m5C, while minimising RNA degradation compared to bisulphite treatment. Despite its advantages, the transformation efficiency of m5C in TAWO‐seq requires optimisation to enhance its applicability in mRNA studies.^30^ TAWO‐seq is a relatively new technique and is not yet widely adopted. However, its ability to identify three known m5C sites in human tRNA, thereby validating the applicability of the approach for analysing real RNA samples.^30^

### 3.3 Immunoprecipitation‐based methods

Immunoprecipitation‐based methods are powerful tools for studying m5C modifications in RNA. These methods use specific antibodies or binding proteins to capture and enrich m5C‐modified RNA, allowing for downstream analyses. Immunoprecipitation‐based approaches are widely used due to their ability to identify m5C modifications across different RNA types and in various biological contexts.

#### 3.3.1 m5C‐RNA immunoprecipitation sequencing

m5C‐RNA immunoprecipitation sequencing (m5C‐RIP‐seq) combines immunoprecipitation with high‐throughput sequencing to identify m5C modifications. RNA is incubated with anti‐m5C antibodies, which specifically bind to m5C residues. The RNA‒antibody complexes are captured using protein A/G beads, and the enriched RNA is purified for sequencing. The success of this method depends on the specificity and affinity of the antibodies, as cross‐reactivity can lead to false positives. While the resolution is limited compared to chemical conversion methods, m5C‐RIP‐seq is highly scalable and has been used to profile m5C across transcriptomes. For example, transcriptome full spectrum analysis was performed on Eimeria tenella mRNA m5C by m5C‐RIP‐seq.^44^

#### 3.3.2 miCLIP

miCLIP provides single‐nucleotide resolution for mapping m5C by utilising cross‐linking of RNA to m5C‐specific antibodies. The cross‐linked sites introduce truncations or mutations during reverse transcription, which are then identified in sequencing reads. This method requires stringent controls and specialised protocols to maintain cross‐link stability. Computational pipelines analyse the mutation patterns to precisely locate m5C sites. miCLIP was used to identify RNA substrates of human NOP2/NUN1.^45^

### 3.4 Third‐generation sequencing

TGS technologies, such as nanopore sequencing and single‐molecule real‐time (SMRT) sequencing, enable direct detection of m5C modifications without the need for chemical conversion. Nanopore sequencing measures disruptions in electrical conductivity as RNA passes through a nanopore, with m5C causing characteristic changes in signal. SMRT sequencing detects m5C by analysing the altered kinetics of reverse transcription, as the enzyme hesitates or changes speed when encountering modifications. These methods allow for transcriptome‐wide analysis of m5C with minimal sample preparation, although they require advanced computational algorithms to interpret the data. For instance, researchers sequenced the complete genome and full‐length transcript of *Saccharomyces cerevisiae* CEN. PK113‐7D.^46^

### 3.5 Predictive models

In the post‐genome era, computational models have emerged as powerful tools for predicting RNA m5C modification sites, supplementing experimental approaches that are often costly, labour intensive and time consuming. These models leverage machine learning algorithms trained on sequence data to predict m5C sites based on sequence context, secondary structures and evolutionary conservation. They also address challenges such as optimising mixed datasets, eliminating false negatives and refining feature extraction methods to enhance prediction accuracy.

A lot of models have been constructed to date for predicting mRNA m5C modifications, including tools such as m5C‐PseDNC,^36^ DeepMRMP,^47^ iRNA‐m5C^35^ and RNA‐m5C‐finder.^48^ Most of these models target human m5C modifications, while a few, such as PEA‐m5C,^4^, ^49^ focus on specific species such as *A. thaliana*. Each tool has unique strengths: DeepMRMP^47^ employs deep learning to predict multiple RNA modifications (e.g., m1A, ψ and m5C) across species, offering higher sensitivity and specificity; m5C‐PseDNC^36^ excels in identifying m5C sites in human samples due to its high sensitivity but has limitations in specificity; RNA‐m5C‐finder^48^ identifies m5C modification sites in various tissues and cell types, including mouse heart cells and human HeLa cells, but lacks cross‐species functionality; iRNA‐m5C^35^ and m5C‐Pred‐SVM^50^ support multi‐species predictions, including *Homo sapiens*, *Mus musculus* and *S. cerevisiae*, while achieving superior accuracy.

Early models faced significant challenges, such as data redundancy and imbalanced datasets, where positive samples far outnumbered negative ones. For instance, iRNA‐m5C‐PseDNC, while offering high sensitivity, suffered from reduced generalizability due to redundant sequences in training data. Subsequent advancements, such as the m5C‐HPCR model,^37^ addressed these issues by using optimised encoding algorithms to enhance performance metrics such as the Matthews correlation coefficient.

More recent developments, such as RNA‐m5C‐Pred^51^ and iMRM,^52^ integrate diverse nucleotide composition features and advanced machine learning techniques such as extreme gradient boosting (XGBoost). These tools not only predict m5C but also incorporate other RNA modifications, providing a comprehensive platform for multi‐modification analysis. For example, iMRM predicts m5C, m6A, m1A, ψ and A‐to‐I modifications in RNA across species, making it cost‐effective and versatile for large‐scale studies.

Despite these advancements, computational models still face challenges, including the need for better species‐specific feature extraction and improved datasets to ensure robust predictions. Current approaches often rely on manually curated training datasets, which may not fully capture the complexity of RNA modifications across species. Species‐specific models remain essential, as m5C modifications exhibit high variability between organisms.

In summary, computational predictive models have become indispensable for identifying potential m5C modification sites. While traditional machine learning methods have been effective, deep learning and hybrid approaches are paving the way for more accurate and scalable predictions. Future efforts should focus on enhancing dataset quality, refining feature extraction methods and improving cross‐species applicability to address the limitations of existing tools. These developments will ultimately enable the analysis of increasingly complex biological samples and experimental conditions, advancing our understanding of RNA methylation mechanisms.

## 4 UNDERLYING MECHANISMS AND BASIC BIOLOGICAL FUNCTIONS OF M5C RNA MODIFICATION

**FIGURE 2:**
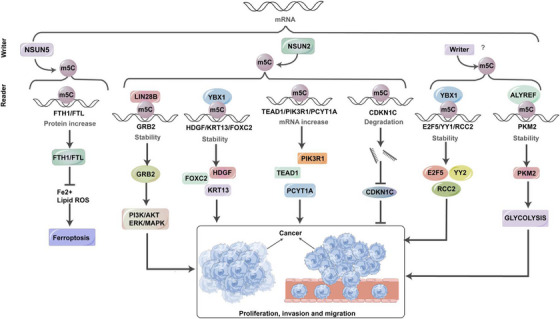
Underlying mechanisms of 5‐methylcytosine (m5C) modulating cancers. The dynamic regulation of m5C modifications in mRNA, mediated by writers (e.g., NSUN2), readers (e.g., YBX1 and ALYREF) and their downstream effects. m5C influences RNA stability, translation and degradation, impacting key cellular processes such as ferroptosis, glycolysis and oncogene expression. Dysregulation of m5C contributes to cancer proliferation, invasion and metastasis, offering potential therapeutic targets in oncology.

RNA m5C undergoes a dynamic and reversible regulation, governed by three key elements: ‘writers’, ‘erasers’ and ‘readers’ (Figure 2).^53^ Within this framework, ‘writers’ denote proteins responsible for facilitating the establishment of methylation patterns. Among the enzymes associated with m5C (RNA Cytosine‐5 Methyltransferases, RCMTs), the NSUN subgroups and the DNMT2 homologue, a DNA methyltransferase, emerge as pivotal catalysts for cytosine‐5 methylation.^18^ Conversely, ‘readers’ represent recognition proteins tasked with binding to and identifying methylated sites. Examples of such proteins encompass the Y‐box‐binding protein 1 (YBX1) and the Aly/REF export factor (ALYREF).^54^ Despite ‘readers’ themselves not participating in catalytic reactions, anomalies in their functions frequently correlate with metabolic abnormalities and diseases.^55^ In comparison, ‘erasers’ play a role in eliminating methylated sites. Noteworthy examples of these erasers include the genes belonging to the TET family and ALKBH1, an ALKB homologue. This intricate interplay establishes a dynamic equilibrium between opposing biological processes (Table 2).^56^

### 4.1 Writers

The methyltransferase RCMTs utilises adenosylmethionine as a methyl group donor to generate m5C by transferring the methyl moiety onto cytosine.^57^ RCMTs are primarily accountable for catalysing the methylation of cytosine‐5, which includes the NSUN methyltransferases and DNMT2.^58^

DNMT2, also referred to as tRNA methyltransferase 1 (TRDMT1), chiefly oversees the methylation processes of tRNA molecules, specifically methylating cytosine at position C38 within the anticodon loop.^59^ Structurally, DNMT2 exhibits a Rossman‐fold enzymatic domain alongside a specific site for S‐adenosyl methionine binding, which is essential for its catalytic activity.^60^ Experimental inquiries have unveiled that the simultaneous deletion of DNMT2 and NSUN2 precipitates a decline in tRNA methylation, inducing hampered protein synthesis and cellular differentiation, ultimately resulting in the demise of experimental mice. Interestingly, deficiencies in either DNMT2 or NSUN2 in isolation failed to elicit discernible impacts. However, a significant decrease in the stable‐state abundance of non‐methylated tRNAs was observed, leading to a marked decrease in the pace of protein synthesis.^61^ Moreover, the involvement of DNMT2 in mRNA methylation and its influence on expression regulation have been extensively documented. DNMT2 deficiency correlates with modifications in mRNA expression and methylation patterns, alongside the hindrance of cell proliferation and migration.^62^ These collective observations underscore the significance of total depletion of cytosine‐5‐tRNA methylation, highlighting its profound influence on the stability of particular tRNAs and underscoring the critical role of tRNA methylation in the intricate process of protein translation.^61^

The NSUN family comprises seven members, denoted as NSUN1‐7, each with specificity towards distinct RNA types.^63^ Notably, NSUN2 stands out as the initial member discovered and extensively investigated among them.^64^ NSUN2 primarily methylates tRNAs, modifying cytosine residues at positions C48‒C50. It also targets mRNAs, especially in 3′‐UTRs with the consensus motif CTCCA, influencing transcript stability and translation. The dynamic spatial distribution of NSUN2 has been linked to the cell cycle in human epidermal cells. It exhibits enrichment in the nucleolus during G1, transitions between nucleoli and nucleoplasm in S phase, localises to the cytoplasm during G2 and resides in centrioles during M phase.^65^ Newly findings have unveiled the methylation of cytosine at positions C48–C50 (m5C) in mitochondrial transfer RNAs (mt‐tRNAs) within mammalian mitochondria. However, suppressing NSUN2 did not markedly impact the stability of mitochondrial tRNAs, suggesting a potentially dispensable role for NSUN2 in mitochondrial tRNA methylation.^66^, ^67^ Additionally, NSUN2 is implicated in biological processes such as cell proliferation and carcinogenesis.^68^ Increased levels of NSUN2 and NSUN2‐mediated m5C have been observed in various types of cancer, including gastric cancer (GC),^69^, ^70^ esophageal squamous cell carcinoma (ESCC),^71^ HCC,^72^ hypopharyngeal squamous cell carcinoma,^73^ prostate cancer (PCa),^74^ cervical cancer,^75^ nasopharyngeal carcinoma^76^ and uveal melanoma.^77^ Moreover, Rennai's research^78^ underscores NSUN2's involvement in regulating RNA methyltransferase activity during human neurocognitive development. In summary, NSUN2 assumes a multifaceted role, impacting plant root morphogenesis, mitochondrial oxidative phosphorylation, tumourigenesis, protein biosynthesis and cellular mitotic progression.^79^ Additionally, it has been documented to regulate HIV replication, Epstein‒Barr virus degradation and epidermal cell differentiation.^80^, ^81^, ^82^

NSUN3, recognised as a tRNA methyltransferase, exerts influence on various biological processes, encompassing tumour advancement,^20^, ^83^, ^84^ infiltration of immune cells^84^, ^85^ and disorders related to mitochondria.^86^ Its increased expression in low‐grade glioma and head and neck squamous cell carcinoma is associated with heightened metastasis, attributed to m5C modification of tRNA at C34 within the anticodon loop.^87^, ^88^

Regarding NSUN4, it operates in the assembly of mitoribosomes, serving as a methyltransferase acting upon 12S rRNA, tRNA and mRNA. This function influences adaptation to elevated temperatures and differentiation into chondrocytes.^89^, ^90^ Furthermore, NSUN4 facilitates the generation of HCC^91^, ^92^ and infiltration of neutrophils.^85^

NSUN5 participates in the methylation of rRNA, introducing m5C3782 in humans and m5C3438 in mice within the 28S rRNA.^93^, ^94^ Its overexpression is linked to tumourigenesis in HCC^95^ and colorectal cancer (CRC)^96^ patients, while deficiency in NSUN5 reduces overall protein synthesis, thus hindering cell proliferation. Notably, NSUN5 is significantly downregulated in patients with tetralogy of Fallot^97^ and Williams‒Beuren syndrome.^98^

Initially acknowledged as a tRNA methyltransferase targeting C72 in the tRNA acceptor stem, NSUN6 has been discovered to possess mRNA methylating activity.^99^, ^100^ It methylates mRNA, primarily focusing on the 3′‐UTR with the consensus motif CTCCA, leading to heightened levels of transcripts and proteins.^101^ The role of NSUN6 in the progression of cancer is convoluted, with evidence suggesting its protective role against triple‐negative breast cancer (TNBC),^102^ pancreatic cancer,^103^ testis cancer,^101^ thyroid cancer^101^ and ovary cancer,^101^ while entailing a hazard factor for CRC.^104^, ^105^ The variability in the impact of NSUN6 across various cancers may arise from its differential expression in immune cells within the tumour microenvironment (TME). Remarkably, NSUN6 exhibits predominant expression in regulatory T cells (Tregs) within TNBC, whereas it is observed in exhausted CD8^+^ T cells, proliferating T cells and myofibroblasts within CRC.^104^ Furthermore, NSUN6 impacts the cell cycle, immune cell infiltration, and the generation of antibody‐secreting plasma cells,^106^ highlighting its multifaceted impact on cancer biology and immune modulation.

NSUN7 carries out methylation of enhancer RNAs, thereby modulating transcription and augmenting the expression of specific mRNAs. This process potentially contributes to adaptive metabolic alterations during starvation in a PGC‐1a‐dependent manner.^107^

### 4.2 Erasers

Erasers dynamically regulate RNA modifications by reversing methylation, thus playing a critical role in modulating RNA function. In the demethylation of m5C, the TET protein family and ALKBH1 have emerged as pivotal enzymes. While the mechanisms of DNA demethylation by these enzymes are well characterised, their roles in RNA demethylation remain an area of active exploration.

The TET family proteins (TET1, TET2 and TET3) primarily catalyse the demethylation of m5C in DNA. This process requires Fe^2^⁺ and α‐ketoglutarate as cofactors and produces intermediates such as 5‐hmC, 5‐formylcytosine and 5‐carboxycytosine.^108^ The demethylation activity of TET1 on mRNA m5C is vital for completing DNA repair and ensuring cell survival following DNA damage.^109^

In mouse embryonic stem cells, the deletion of TET2 significantly reduces the levels of 5‐hmC in tRNAs, while TET2 overexpression increases 5‐hmC levels and decreases m5C levels. These findings indicate that TET2 can oxidise m5C in tRNAs, contributing to dynamic regulation. Moreover, in vitro experiments suggest that this oxidative process may enhance translational efficiency, highlighting a potential link between TET2‐mediated RNA demethylation and protein synthesis. Despite these findings, the oxidation efficiency of RNA m5C by TET proteins is considerably lower than that of DNA, suggesting distinct catalytic mechanisms for RNA and DNA substrates.^110^, ^111^

TET3, in particular, has been implicated in increasing RNA 5‐hmC levels, but its efficiency in oxidising RNA m5C remains low.^112^ Furthermore, the structural basis of TET‒RNA complexes is still unclear, which limits the understanding of their substrate specificity and catalytic mechanisms. However, some studies suggest a potential correlation between elevated TET3 expression and poor prognosis in PCa patients.^16^ Cellularly, the constituents of the TET family demonstrate disparate spatial distributions; TET3 is present in both the nucleus and cytoplasm, whereas TET1 and TET2 are predominantly localised in the nucleus.^113^

ALKBH1 serves as a demethyltransferase for both RNA and DNA substrates.^114^ Studies suggest that ALKBH1 participates in converting m5C RNA modifications into either 5‐hmC or 5‐formylcytidine (f5C) RNA modifications.^115^ In HEK293T cells, ALKBH1 deletion results in a marked reduction of f5C modifications in mt‐tRNAs, leading to impaired mitochondrial translation and respiratory function. These findings underscore the essential role of ALKBH1‐mediated m5C demethylation in maintaining mitochondrial activity.^115^ Deficiency in ALKBH1 results in a significant impairment in mitochondrial translation and oxygen consumption, underscoring the substantial regulatory role of ALKBH1 in RNA m5C metabolism concerning mitochondrial function. Furthermore, ALKBH1 exhibits specificity in targeting a histone dioxygenase‐histone H2A.^116^ Beyond its impact on mt‐tRNAs, ALKBH1 may influence other RNA types by modulating their stability and translational efficiency.^117^ This broad functional spectrum highlights the importance of ALKBH1 in cellular metabolism and suggests a potential link to pathological states, including mitochondrial disorders and cancer progression. Future research should focus on elucidating ALKBH1's substrate range and its interplay with cellular metabolic pathways.

### 4.3 Readers

The biological roles of RNA modification are predominantly linked to the proteins with which it forms associations. Such proteins, termed ‘readers’, interpret the epigenetic code imparted by m5C modifications, mediating their downstream effects. Proteins binding to RNA ‒m5C, such as ALYREF and YBX1, function as code interpreters, recognising and binding to ‒m5C sites.^114^

ALYREF, identified as the primary mRNA‐reading protein in the nucleus, discerns m5C within the viral RNA transcript localised in the nucleus and facilitates its translocation to the cytoplasm.^23^ ALYREF binding to m5C facilitates the nuclear export of these transcripts to the cytoplasm, ensuring proper RNA trafficking and translation. This process has been shown to significantly influence cellular processes such as apoptosis, growth and energy metabolism, particularly in cancer cells. Across various cancer scenarios, the expression levels of ALYREF exhibit correlations with patient survival. Elevated ALYREF expression, particularly observed in BC, is linked to unfavourable survival outcomes. In BC cells, ALYREF exerts influence over cellular processes such as growth, apoptosis and mitochondrial energy metabolism, primarily through its interaction with the lncRNA NEAT1.^118^ This underscores the intricate interplay between RNA modifications, binding proteins and cellular functions in cancer contexts. Moreover, ALYREF has a pivotal role in the regulation of mRNA and lncRNA degradation within the nucleus. Research carried out in HeLa and HEK293 cells has demonstrated that this degradation process involves the nuclear exosome and is facilitated by the cofactor hMTR4, which recruits the exosome to its designated targets. Intriguingly, investigations by Fan et al.^119^ and collaborators have unveiled a competitive interaction between hMTR4 and ALYREF in their binding to ARS2. This competitive dynamic significantly influences the destiny of an RNA molecule. When ARS2 binds with ALYREF, the latter is mobilised to the RNA. If the RNA is stabilised by ribonucleoprotein (RNP) factors, it undergoes translocation into the cytoplasm. Conversely, if ALYREF fails to engage with ARS2 or RNP factors, hMTR4 binds to ARS2 on the RNA, initiating the recruitment of the exosome for degradation.^119^ This intricate interplay underscores the regulatory intricacy orchestrated by ALYREF in RNA processing and cellular equilibrium.

YBX1 exhibits versatility by participating in various cellular processes, including translational repression, RNA stabilisation, mRNA splicing, DNA repair and transcriptional regulation.^120^ Through structural and thermal analyses, it has been determined that YBX1 can recognise m5C within its cold‐shock domain (CSD), particularly through the indole ring of W65.^121^ This protein selectively targets oncogenes that contain m5C, such as heparin‐binding growth factor (HDGF), thereby increasing their stability and promoting cancer progression by recruiting the well‐established mRNA stability maintenance factor ELAVL1.^122^ Recent investigations have revealed that YBX1 can identify approximately 87.8% of mRNAs modified by m5C. Depleting YBX1 during early zebrafish embryo development leads to a significant reduction in the expression of 397 maternal mRNAs bearing m5C. This observation implies a critical role for YBX1‐mediated m5C modifications in governing the clearance of maternal genes during the transition from maternal to zygotic control.^123^ In Drosophila, the YBX1 homologue, known as Ypsilon Schachtel (YPS), also serves as an RNA m5C reading protein.^124^ Within the Drosophila ovary, YPS contributes to the homeostasis, proliferation, and differentiation of germline stem cells (GSCs), relying on the binding of m5C‐containing RNAs. Intriguingly, like YBX1 in humans and zebrafish, the highly conserved CSD of YPS also harbours the m5C binding site. Mutations in this site can lead to defective development of GSCs, suggesting that RNA m5C modification plays a role in influencing the development of adult stem cells.^125^

## 5 THE ROLE OF M5C RNA MODIFICATION IN CANCERS

**FIGURE 3:**
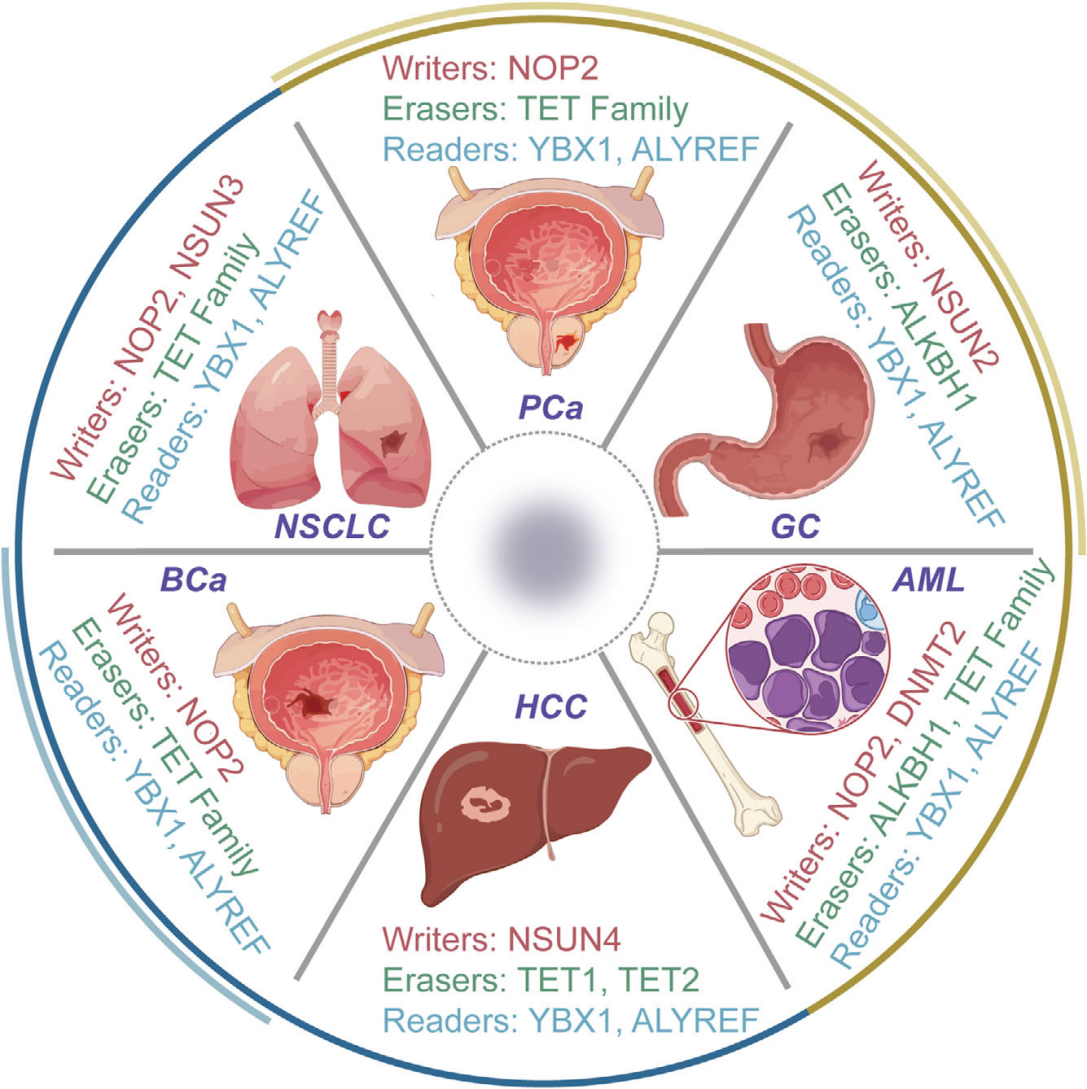
Role of 5‐methylcytosine (m5C) in cancers. Role of m5C regulators, writers, erasers and readers in various cancer types, including non‐small‐cell lung cancer (NSCLC), prostate cancer (PCa), gastric cancer (GC), acute myeloid leukaemia (AML), bladder cancer (BCa) and hepatocellular carcinoma (HCC).

RNA m5C modification has become a significant biological phenomenon in various pathological conditions, particularly in neoplastic disorders such as cancer.^126^ Changes in the expression profiles of m5C writers, readers and erasers have a noticeable impact on the development of tumours, progression of malignancy and metastasis by causing alterations in both mRNA and non‐coding RNAs at the levels of expression and transcription. This study aims to provide a comprehensive understanding of the molecular changes induced by m5C within cancer cells, as explored so far (Figure 3).

To present a detailed exploration, we have focused on cancers where m5C‐related mechanisms have been most extensively studied, such as BCa, HCC, leukaemia, GC, PCa, BCa and lung cancer. These cancers exemplify the diverse ways in which m5C influences tumour biology, providing critical insights into the regulatory roles of m5C modification. For other cancers, such as pancreatic cancer, CRC and cholangiocarcinoma, which have emerging but comparatively limited data, we have provided concise discussions to highlight significant findings while maintaining a balanced presentation across cancer types.

### 5.1 BCa

The exploration of RNA m5C modification landscape offers valuable insights into comprehending BCa development. Within cancer tissues, the heightened expression of NSUN2 and YBX1, functioning as the ‘writer’ and ‘reader’ of m5C, respectively, contributes to tumour formation by upholding mRNA stability. This aberration strongly correlates with an unfavourable prognosis for BCa patients.^23^ To elaborate, NSUN2 methylates the mRNA of the oncogenic gene HDGF,^127^, ^128^ concentrating significantly in its 3′‐UTR. Subsequently, the RNA‐binding protein YBX1 recognises these m5C‐methylated sites, facilitating the recruitment of ELAVL1, thereby ensuring the stability of HDGF mRNA. This intricate process assumes a central role in the oncogenic function of YBX1 in the advancement of BCa by triggering the activation of HDGF.^53^ During clinical scenarios, heightened co‐expression of NSUN2, YBX1 and HDGF signifies the poorest survival outcomes. Additionally, the presence of m5C‐modified mRNAs in tumour‐related pathways, including the Phosphoinositide 3‐Kinase‐Protein Kinase B Signaling Pathway (PI3K‐AKT) and Extracellular Signal‐Regulated Kinase‐Mitogen‐Activated Protein Kinase (ERK‐MAPK) signalling pathways, suggests that m5C hypermethylation may instigate tumourigenesis by activating oncogenic signalling pathways.^129^

### 5.2 HCC

Previous investigations have established a clear correlation between elevated m5C levels and the progression, migration and malignancy of HCC.^130^ Recent research has focused on lncRNAs in relation to HCC. For example, during the course of HCC, there is a significant increase in the expression of m5C‐associated genes, embracing NSUN2,^72^ NSUN4,^91^ NSUN5^95^ and ALYREF.^131^ NSUN2 promotes HCC cell growth by stabilising the mRNA of fizzy‐related‐1 (FZR1),^132^ a critical regulator of mitosis and the G1 phase of the cell cycle.^133^ Furthermore, NSUN2 introduces m5C modifications on H19 lncRNA, allowing it to compete with MYC mRNA for binding to G3BP1, resulting in MYC accumulation and driving HCC cell development.^72^ These mechanisms collectively drive HCC proliferation, migration and poor differentiation. The elevated expression of m5C regulators, specifically NSUN4 and ALYREF, is inversely associated with the survival outcomes of HCC patients, highlighting their importance as diagnostic and prognostic biomarkers for HCC.^92^ A comprehensive bioinformatics analysis has shown a positive correlation between NSUN5 overexpression and enhanced ribosomal activities and protein synthesis in HCC cells.^95^ Dysregulation of ALYREF is associated with the irregular control of the cellular division cycle and mitotic processes in HCC cells, potentially promoting HCC through the stimulation of eIF4A3 expression.^92^, ^131^ Therefore, antagonists directed towards ALYREF and eIF4A3, exemplified by miR‐4666a‐5p and miR‐6124, are burgeoning as auspicious contenders for therapeutic intervention. Additionally, the modulation of m5C in circular RNA has a vital role in the development about HCC.^134^ Furthermore, modifications in the TME and the infiltration of immune cells also play a role in m5C‐mediated HCC progression.^135^ Recently, researchers are actively exploring novel approaches to predict prognosis in HCC patients. Speculative models have been constructed based on m5C‐related modulators, including members of the NSUN family, TET1 and YBX1.^136^ These insightful findings have the potential for significant clinical implications in the understanding and management of HCC.

### 5.3 Leukaemia

RNA m5C alteration and the impact of RCMTs have been elucidated to exert an effect on the responsiveness to pharmacotherapeutics and resilience, not solely in neoplasms of a solid nature but also in the context of leukaemia. These molecules engage with various partners to control diverse chromatin configurations. Specifically, NSUN1 collaborates with BRD4 to establish a distinctive NSUN1/BRD4/RNA‐pol‐II CTD‐S2P complex in leukaemia cells resistant to 5‐AZA. This intricate formation directly engages with RNA‐pol‐II CTD‐S2P, fostering the creation of a chromatin framework resilient to 5‐AZA, thereby fostering resistance to this medication in leukaemia.^137^ Conversely, NSUN3 and DNMT2 exhibit antagonistic impacts on 5‐AZA‐responsive leukaemia cells. The RNA‐binding protein hnRNPK directly engages with RCMTs (NSUN3 and DNMT2), pivotal transcriptional regulators (GATA1 and SPI1/PU.1) and CDK9/P‐TEFb to enlist RNA polymerase II and establish a distinct assembly, thereby fostering the formation of a chromatin configuration susceptible to 5‐AZA.^53^ In a clinical trial utilising bone marrow (BM) samples from both 5‐AZA‐resistant and 5‐AZA‐sensitive leukaemia cases, elevated levels of m5C mRNA were observed in the 5‐AZA‐resistant specimens. The expression levels of hnRNPK, NSUN1 and BRD4 were also linked to leukaemia progression and implicated in both 5‐AZA resistance and cancer advancement. These observations underscore the significant roles of hnRNPK, NSUN1 and BRD4 in the mechanisms underlying 5‐AZA resistance and leukaemia progression. Separately, TET2 deficiency in acute myeloid leukaemia increases the expression of Tetraspanin 13 (TSPAN13) by enhancing m5C modification in its mRNA and stabilising it through YBX1, thereby activating the CXCR4/CXCL12 signalling pathway to promote leukaemia stem cell homing to the BM niche and enhance self‐renewal.^138^ Furthermore, TET2 depletion impairs m5C oxidation and antagonises the MBD6‐dependent deubiquitination of Lys119 of histone H2A (H2AK119ub), leading to open chromatin and increased transcription in stem cells. Targeting MBD6 may offer a therapeutic strategy for TET2‐mutant leukaemia.^139^ Consequently, targeting m5C could represent a promising therapeutic strategy for haematologic malignancies.^137^

### 5.4 GC

The involvement of m5CRNA modification in GC generally takes on an oncogenic role, as elevated m5C levels are indicative of an unfavourable prognosis and a diminished overall survival rate.^140^ Within this context, NSUN2 emerges as the principal oncogenic m5C methyltransferase associated with GC.^130^, ^141^ Its specific influence extends to the methylation status of mRNAs linked to pivotal genes, including phosphoinositide‐3‐kinase regulatory subunit 1 (PIK3R1), phosphate cytidylyltransferase 1 choline‐alpha (PCYT1A), Forkhead box protein C2 (FOXC2)^142^ and p57Kip2, a cyclin‐dependent kinase (CDK) inhibitor.^141^, ^143^, ^144^ Following m5C modification and subsequent binding to m5C readers, notably exemplified by YBX1, there is a noticeable augmentation in the transcriptional activity of PIK3R1, PCYT1A and FOXC2 mRNA. Conversely, the tumour‐suppressive p57Kip2 mRNA undergoes destabilisation due to m5C modification in the 3′‐UTR.^145^ As a result, heightened levels of NSUN2 in GC patients contribute to increased proliferation, migration and invasion of cancerous cells. The activation of NSUN2, facilitated by the small ubiquitin‐like modifier (SUMO)‐2/3, involves a direct interaction that stabilises NSUN2 and facilitates nuclear transport. This activated state, in turn, propels the progression of GC. Furthermore, the oncogenic interplay between NSUN2 and FOXC2 mRNA can be facilitated by the lncRNA FOXC2‐AS1 (FOXC2 antisense RNA 1). This intricate network of molecular events underscores the oncogenic role of NSUN2 and its downstream targets in the pathogenesis of GC.^141^

### 5.5 PCa

Several prognostic models have been developed for PCa patients based on m5C modulators or m5C‐related lncRNAs.^146^ Experimental findings consistently demonstrate that elevated levels of NSUN2, YBX1 and TET3 are associated with an unfavourable prognosis. NSUN2, through its interaction with YBX1, post‐transcriptionally modifies androgen receptor (AR) mRNA with m5C, leading to increased stability and translation of AR mRNA. This molecular interplay highlights the collective influence of these regulatory elements on clinical outcomes, emphasising the critical role of NSUN2‐mediated m5C modification in AR regulation and its impact on disease prognosis.^147^ Notably, a reciprocal activation loop exists, as AR positively modulates NSUN2 at the transcriptional tier. This bidirectional regulation creates a feedback mechanism, suggesting a tightly interconnected relationship between AR and NSUN2 expression. Furthermore, elevated NSUN2 expression correlates with diminished responsiveness to chemotherapy and attenuated immune cell infiltration, highlighting the multifaceted impact of NSUN2 in cancer. This implicates NSUN2 not only in AR regulation but also in the modulation of therapeutic responsiveness and the TME.^148^ Additionally, the m5C reader, ALYREF, has been pinpointed as a prognostic biomarker and a plausible therapeutic target for PCa. The m5C signature exhibits potential as a novel instrument for prognosticating patient outcomes across various molecular subtypes and for steering personalised treatments contingent upon therapeutic responses.^149^

### 5.6 BC

The modification of m5C RNA exhibits both advantageous and detrimental impacts on the progression of BC. Recent investigations have pinpointed NSUN2, ALYREF and DNMT3B as factors posing risks.^102^, ^150^ Conversely, NSUN5, NSUN6, TET2 and DNMT2 have emerged as protective elements.^151^ Mechanistically, in TNBC, both NSUN2 and NSUN6 play critical roles in tumourigenicity and the tumour immune microenvironment. Additionally, the upregulation of NSUN2 and NOP2 is significantly associated with shorter disease‐free survival in BC patients.^130^ Interestingly, NSUN6 may promote bone metastasis. Specifically, HER3 is phosphorylated by the tyrosine kinase (RTK)‐like orphan receptor 1 (ROR1), which recruits NSUN6 to methylate MST1, thereby modulating its kinase activity and activating YAP. The nuclear accumulation of YAP subsequently stimulates the expression of genes associated with tumour proliferation and bone metastasis.^152^ The amplification of ALYREF, detected in both mRNA and protein expressions, has been linked to its interaction with the promoter region of the oncogenic NEAT1 lncRNA, thereby facilitating its transcriptional augmentation.^118^ Moreover, through thorough enrichment analysis, prospective targets of DNMT3B have been delineated, encompassing vascular endothelial growth factor A and enhancer of zeste homologue 2 (EZH2). Additionally, the modification activities of NSUN5, TET2 and DNMT2 have been discerned to exert influence over three specific lncRNAs, namely, AP005131.2, AL121832.2 and LINC01152, rendering them as defensive shields against BC.^151^ This comprehensive scrutiny illuminates the intricate molecular pathways involving ALYREF, DNMT3B, NSUN5, TET2 and DNMT2 in sculpting the regulatory framework of lncRNAs associated with BC.

### 5.7 Lung cancer

The heightened presence of m5C modification within RNA, particularly evident in circulating tumour cells extracted from lung cancer patients when contrasted with whole blood cells, underscores a unique signature linked with the onset of tumourigenesis.^153^ Various indications suggest the involvement of RNA cytosine methyltransferases NSUN2^87^, ^154^ and DNMT2^155^ in tumourigenesis, albeit their precise mechanisms remaining elusive. NSUN2, functioning as a writer for m5C, emerges as a direct target of MYC, a renowned modulator of tumour cell proliferation, initially identified for its upregulation in malignant skin tumours.^156^ Similar to MYC, NSUN2 was also highly expressed in various tumours.^157^, ^158^ Additional inquiries are warranted to unravel the plausible implications of m5C modification in the progression of lung cancer, particularly the potential contributions of NSUN2, potentially through interactions with lncRNAs. Notably, the heightened expression of H19 lncRNA, observed in numerous adult malignant lung cancer tumours, has been linked to NSUN2‐mediated m5C modification, resulting in H19 stabilisation and subsequent pro‐oncogenic effects.^72^ This association emphasizes the complex interplay between NSUN2, m5C modification and lncRNA regulation in the context of lung cancer, necessitating further exploration to uncover their precise mechanistic contributions.

### 5.8 Other cancers

Apart from the aforementioned cancer types, the oncogenic mechanisms of m5C in other malignancies remain poorly understood. In individuals diagnosed with pancreatic cancer, NSUN2 emerges as a pivotal controller influencing both the onset of pancreatic tumourigenesis and the differentiation of epithelial cells through its involvement in mRNA methylation.^159^ Conversely, NSUN6 and DNMT3A have exhibited inhibitory impacts on pancreatic cancer, effectively curbing the proliferation of malignant cells.^103^ NSUN6 may regulate pancreatic cancer tumour growth by modulating CDK10.^130^ However, further elucidation is warranted regarding the precise mechanisms orchestrating this inhibition.^160^

Among CRC patients, heightened levels of regulators associated with m5C, including NSUN5, NSUN6, ALYREF and YBX1, have been noted by researchers.^96^ Notably, the diagnostic potential of m5C levels in immune cells within peripheral blood surpasses that of conventional blood tumour biomarkers, suggesting a role for m5C modification in impeding the infiltration of immune cells into tumours.^161^

In individuals suffering from cholangiocarcinoma, the presence of m5C‐modified functional lncRNA NKILA (NF‐kappa B interacting lncRNA), recognised and stabilised by YBX1, correlates with an advanced TNM stage and unfavourable prognosis.^162^ Similarly, glioma patients manifest heightened expression of m5C‐related genes, including NSUN1‐5, NSUN7, DNMT1, DNMT3B and YBX‐1, excluding NSUN6.^83^ In the context of glioblastoma, a formidable subtype of diffuse glioma, the heightened expression of ALYREF undertakes an oncogenic function by triggering the Wnt/β‐catenin signalling pathway and enhancing the resilience of MYC mRNA.^163^, ^164^

Among neuroblastoma patients, the m5C reader ALYREF forms a nuclear coactivator complex alongside MYCN, thereby prompting USP3 transcription and fostering neuroblastoma tumourigenesis.^165^ Meanwhile, in cases of ESCC, NSUN2 fosters disease progression and chemoresistance by amplifying the expression of TIGAR (TP53‐induced glycolysis regulatory phosphatase)^166^ and growth factor receptor bound protein 2 (GRB2).^71^ The NSUN2‐dependent LIN28B pathway exerts a positive influence on GRB2, culminating in m5C modification of GRB2 mRNA, thereby indirectly stimulating the PI3K/AKT and ERK/MAPK signalling cascades.

NSUN2 and YBX1 play important roles in catalysing and recognising methylation sites, leading to the methylation of KRT13 mRNA and facilitating translational initiation.^167^ During ovarian cancer patients, YBX1 acts as a regulator that influences the expression of various downstream targets, including CD44, thereby increasing chemoresistance.^168^ On the other hand, NSUN6, a methyltransferase, is involved in RNA m5C modification and has a suppressive effect on testis, thyroid and ovary cancers.^101^ Additionally, the NSUN2‐driven RNA m5C modification is linked to the modulation of uveal melanoma cell proliferation and motility. However, the exact mechanisms underlying these effects are still unknown.^77^ This comprehensive explanation highlights the complex regulatory roles of m5C‐related factors in different types of cancer, providing valuable insights into the molecular mechanisms of tumourigenesis and progression.

Notably, recent research has uncovered a significant interaction between glucose and the amino acids of NSUN2, facilitating its oligomerisation and activation.^169^ Activated NSUN2 plays a crucial role in maintaining m5C RNA methylation, stabilising TREX2, and limiting the accumulation of membrane‐associated DNA while suppressing cGAS/STING activation. This, in turn, promotes tumourigenesis and contributes to resistance against PD‐L1 immunotherapy. The study elucidates the pivotal role of glucose in tumourigenesis and immune therapy resistance. Glucose, through the modulation of NSUN2 activity, exerts influence over global m5C RNA methylation and the accumulation of membrane‐associated DNA, ultimately fostering tumour development and immune therapy resistance. These findings provide a novel perspective on the molecular mechanisms underlying tumourigenesis, offering insights into the intricate interplay between glucose metabolism, NSUN2 activation and the modulation of anti‐tumour immune responses.

RNA m5C modification has emerged as a pivotal epigenetic mechanism influencing gene regulation across various cancer types. Its role extends beyond gene expression modulation to impacting tumourigenesis, progression, metastasis, immune evasion and therapeutic resistance. Further research is required to elucidate the molecular mechanisms underpinning m5C regulation in cancers and to harness its diagnostic and therapeutic potential. Understanding these processes can pave the way for innovative cancer treatments targeting RNA modifications.

## 6 CONCLUSIONS AND PERSPECTIVES

Epigenetic modifications are increasingly recognised for their role in biological processes, with recent focus on nucleic acid methylation. This review specifically examines the relatively less explored RNA m5C modification in tumour cells. While m5C methylation has received less attention compared to its counterpart, m6A methylation, it remains an important aspect of epigenetic modification. Several writers and readers of RNA m5C have been discerned, exerting influence over a myriad of biological phenomena. DNMT2, concomitant with NSUN2, NSUN3 and NSUN6, focalise on tRNAs, contributing to their fragmentation and influencing energy metabolism.^20^, ^61^, ^66^, ^67^, ^83^, ^84^, ^99^, ^100^ NSUN1, NSUN4 and NSUN5 selectively engage with rRNA, shaping ribosomal metabolism.^45^, ^89^, ^93^ Regarding mRNA, DNMT2 and NSUN2 emerge as the principal artisans, with m5C modification in mRNA pivotal to cellular processes encompassing growth, differentiation, motility and RNA alteration.^62^, ^65^ Further exploration in this domain is merited. NSUN2 and NSUN7 collaborate with non‐coding RNAs to govern cellular metabolism.^170^ Writers of RNA m5C, such as NSUN enzymes, collaborate with readers like YBX1 and ALYREF to enhance mRNA transportation and stability.^114^ In contrast, YTHDF2 binds to m5C‐modified rRNA, similar to m6A modification.^171^ However, comprehensive erasers for RNA m5C are still elusive. DNA demethylases TETs are implicated in potential cascade oxidations, suggesting a mechanism for RNA demethylation in the m5C region. The co‐functionality of methylation components in DNA and RNA, observed in m6A, m5C, 5‐hmC and m1A modifications, warrants investigation into whether they induce methylation or demethylation and the associated mechanisms. Despite this gap, various types of RNA methylation collaborate to regulate disease development. For instance, the collaborative action of NSUN2‐mediated m5C with METTL‐mediated m6A serves to bolster p21 expression, thereby exemplifying their involvement in the onset and advancement of glioma.^172^

The pivotal involvement of RNA m5C has been recognised in diverse pathological conditions, encompassing disorders in energy metabolism,^173^ afflictions of the hematopoietic system,^139^ viral infections,^174^ and notably, the progression of malignancies.^20^ The expression of RNA m5C writers and readers has been found to be significantly associated with poor prognosis and TNM classification in neoplasms, particularly affecting tumourigenesis and metastasis in different tissues such as the brain, lung,^154^ breast^130^ and prostate.^74^ Cytokines such as HDGF, TGF‐β, FGF2 and G3BP1, which are recognised tumour promoters, contribute to cell migration and metastasis through m5C modification, highlighting the connection between RNA m5C levels and cancer cell mobility. The presence of elevated RNA m5C levels in circulating tumour cells further underscores its potential as a prognostic marker for cancer.^153^ The discovery of m5C in circulating DNA highlights its potential as a biomarker for cancer prognosis. While RNA m5C and DNA m5C modifications share common methyltransferases and may both play roles in tumour biology, the direct connection between these modifications requires further investigation to establish mechanistic links or clinical relevance.

RNA m5C modification is a potential therapeutic target, particularly in gene therapy for diseases induced by RNA m5C and cancer progression. Modifying key RNA m5C methylases, such as NSUN2, through gene sequence alterations can reduce RNA methylation, thereby preventing disease onset or reversing cancer development. While gene therapy lacks specificity, the development of well‐designed molecular inhibitors that target specific RNA m5C writers^175^ shows promise in countering disease progression, taking inspiration from the successful use of m6A component inhibitors in cancer therapy. Additionally, exploring upstream molecules that regulate the functional activity of RNA m5C provides an alternative treatment approach. For instance, the phosphorylation of NSUN2 at Ser‐139 by Aurora kinase B has been shown to result in reduced RNA methylation levels. This suggests that protein‐level regulation is feasible.^176^ To fully harness the potential of RNA m5C modification in clinical applications, further research into molecular structures and functional pathways is imperative. Tables 1, 2

**TABLE 1:** Current techniques for 5‐methylcytosine (m5C) detection.

Detection methods	Subdivision	Advantages	References
Physicochemical methods	HPLC	Remarkable sensitivity, capable of detecting substances at the nanogram level.	^26^
	MS	Useful for direct RNA sequencing and transcriptomic analysis, allowing for the detection of modification types.	^39^
	LC‒MS/MS	Suitable for detecting low‐abundance modifications.	^41^
	Top‐down MS	Particularly effective in detecting, localising, and relatively quantifying RNA modifications.	^177^
	NAIL‐MS	Available for the assessment of the external environment's influence on the apparent transcriptome.	^178^
	Nano‐LC‒MS/MS/MS	Heightened chemical specificity and superior sensitivity.	^11^
	2D‐HELS MS Seq	Increased precision and versatility in the detection of various modifications.	^179^
Chemical conversion methods	RNA‐BisSeq	Gold standard for detecting m5C modifications. Achieving single‐base resolution in recognising cytosine methylation sites.	^29^
	TAWO‐seq	Minimising RNA damage compared to RNA‐BisSeq and avoiding false positives caused by incomplete transformation of unmodified cytosines.	^30^
	UBS‐seq	Enabling the detection of numerous sites with low modification ratios.	^31^
Immunoprecipitation	m5C‐RIP‐seq	Available for the detection of m5C modifications without altering the RNA sequence or requiring RNA‐modifying enzymes.	^32^
	5‐AZA‐seq and miCLIP	Effectively addressing the non‐specific binding issues of MeRIP‐seq and accurately locates individual nucleosides.	^180^
TGS	NGS	Accurately identifying modified nucleosides in the transcriptome.	^181^
	SMRT	Eliminating the need for RNA recombination or qPCR amplification, enhancing continuity and integrity.	^34^
Predictive models	PEA‐m5C	A hybrid model for quickly and accurately identifying m5C sites from non‐m5C sites in *Homo sapiens* RNA.	^49^
	Pm5CS‐Comp‐mRMR	Utilising an mRMR algorithm that offers heightened sensitivity compared to m5C‐PseDNC.	^182^
	m5C‐PseDNC	Highly accurately predictive performance, effectively identifying the positions of m5C in DNA or RNA sequences.	^35^
	iRNA‐m5CPseDNC	Highest overall accuracy, particularly in detecting m5C sites in human samples, with notable sensitivity.	^36^
	m5C‐HPCR	Performance metrics and Matthews correlation coefficient, outperformed those of iRNA‐m5C‐PseDNC.	^37^
	RNA‐m5C‐Pred	Improved specificity and sensitivity compared to models developed before 2019.	^51^
	iRNA‐PseColl	Identifying methylation sites for three RNA modifications.	^183^
	RNA‐m5C‐finder	Recognising m5C modification sites in various tissues.	^48^
	iRNAm5C	Detection for m5C modification sites in four species.	^35^
	m5CPred‐SVM	Outperforming existing methods in detecting m5C sites in specific species.	^50^
	DeepMRMP	Efficiently predicting multiple modification types across different species.	^47^
	iMRM	Predicting various RNA modifications in *H. sapiens*, *Mus musculus* and *Saccharomyces cerevisiae*.	^52^

Abbreviations: HPLC, high‐performance liquid chromatography; LC‒MS/MS, liquid chromatography‒tandem mass spectrometry; MS, mass spectrometry; NGS, next‐generation sequencing; SMRT, single‐molecule real‐time; TGS, third‐generation sequencing;

**TABLE 2:** Common writers, readers and erasers of 5‐methylcytosine (m5C) modification.

Types	Proteins	Substrate	Target sites	Cellular functions	Mechanisms	References
Writers	DNMT2	tRNA‐Asp	C38	Protein synthesis and cellular differentiation		^60^
				Cell proliferation and migration		^184^
	NSUN1	28S rRNA	Conserved cytosines	Ribosome biogenesis	Modulates pre‐rRNA processing through interaction with the 5′‐ETS region of the pre‐rRNA transcript, forming a non‐catalytic complex in conjunction with box C/D snoRNAs.	^45^
				HIV‐1 viral latency	Competes with HIV‐1 Tat protein to interact with HIV‐1 TAR RNA.	^185^
				Cell growth		^186^
	NSUN2	tRNA, mRNA	C34, C48 (tRNA); UTRs, CDS (mRNA)	Synaptic signalling at prefrontal cortex pyramidal neurons and contextual fear memory		^187^
				Cell proliferation and migration	m5C‐methylates GRB2 and CD44.	^68^
				Progression of GC	m5C‐methylates PIK3R1, PCYT1A and FOXC2 mRNAs; represses p57Kip2 by destabilising its mRNA in a m5C‐dependent manner.	^145^, ^188^
				Progression of ESCC	m5C‐methylates GRB2 via LIN28B‐dependent way, thus activating PI3K/AKT and ERK/MAPK signalling pathway.	^71^, ^166^
				Progression of HCC	m5C‐methylates H19 lncRNA, leading to MYC stimulation; modulates Ras signalling pathway and cell cycle.	^189^
				Progression of HPSCC	m5C‐methylates TEAD1 mRNA, thus upregulating its expression.	^190^
				Progression of PCa	m5C‐methylates and stabilises AR mRNA.	^147^
				Progression of cervical cancer	m5C‐methylates KRT13 mRNA, enhancing its binding with m5C reader YBX1.	^167^
				Progression of NPC	Negatively regulates immune cell infiltration in TME.	^191^
				Progression of uveal melanoma		^77^
				IL‐17A secretion of T cells	m5C‐methylates IL‐17A mRNA in T cells.	^192^
				p21 expression under conditions of oxidative stress‐induced cellular senescence	m5C‐methylates p21 mRNA at the 3′‐UTR.	^172^
				ALYREF's nuclear‐cytoplasmic shuttling, RNA‐binding affinity and associated mRNA export		^8^
	NSUN3	mt‐tRNA	C34 in anticodon loop	Low‐grade glioma development		^83^
				Development of HNSCC	Promotes tumour progression by regulating immune cell infiltration.	^84^
				Mitochondrial mRNA translation, thus promoting metastasis		^87^
				CD8+ T cells and M2 macrophages infiltration		^85^
				Mitochondrial regulation		^86^
	NSUN4	12S rRNA, tRNA	Multiple	Facilitates mitoribosomal assembly	m5C‐methylates 12S rRNA and interacts with MTERF4.	^89^
				Facilitates adaptation to higher temperatures	Ensures translation efficiency of UUG‐rich transcripts and fertility.	^90^
				Chondrogenic differentiation	m5C‐methylates the 3′‐UTR of Sox9 mRNA.	^193^
				Progression of HCC		^91^
	NSUN5	28S rRNA	m5C3782 (humans)	Protein synthesis and cell proliferation		^93^
				Progression of HCC	Strengthens ribosome functions and global protein translation.	^95^
				Progression of CRC		^96^
	NSUN6	tRNA, mRNA	C72 in tRNA, 3′‐UTRs	Suppression of TNBC	Potentially regulates infiltration of CD4^+^ T cells.	^102^
				Suppression of pancreatic cancer	Promotes tumour‐suppressive CDK10.	^103^
				Suppression of testis, thyroid and ovary cancers	Promotes higher expression and translation levels of m5C‐methylated mRNAs.	^101^
				Progression of CRC		^105^
				Cell cycle dysfunction		^194^
				Infiltration of B cells and CD8^+^ T cells		^104^
				Formation of antibody‐secreting plasma cells		^106^
	NSUN7	eRNA	Enhancer regions	transcriptional coactivator function of PGC‐1a		^107^
Erasers	TET1	DNA, RNA (potential)	DNA: CpG sites RNA: unclear, low efficiency	Proper completion of DNA repair and survival of cells after DNA damage	Mediates mRNA m5C‐demethylation, thus promoting mRNA‐dependent recombination.	^109^
	TET2	DNA, RNA (tRNA)	DNA: CpG sites RNA: tRNA, m5C sites	Conversion of m5C into 5‐hmC		^110^
				Promotion of low‐grade glioma		^83^
				Suppression of ccRCC		^195^
				Inhibition of ovarian cancer		^196^
				Inhibition of prostate adenocarcinoma	Potentially promotes immune cell infiltration.	^197^
	TET3	DNA, RNA (potential)	DNA: CpG sites RNA: possible mRNA targets	Promotion of PCa		^115^
	ALKBH1	RNA (tRNA, mt‐tRNA), DNA	RNA: mt‐tRNA C34 (anticodon loop), m5C DNA: n/a	Converting m5C RNA modifications into either 5‐hmC or 5‐formylcytidine RNA modifications		^115^
Readers	ALYREF	mRNA; lncRNA	m5C‐modified sites in nuclear mRNA and viral RNA transcripts; NEAT1	Progression of HCC	Promotes eIF4A3 expression; disrupts cell cycle and mitosis regulation.	^92^, ^198^
				Progression of glioblastoma	Stabilises MYC mRNA; activates the Wnt/β‐catenin signalling pathway.	^163^
				Progression of glioma		^83^
				Progression of neuroblastoma	Forms a nuclear coactivator complex with MYCN to stimulate USP3 transcription.	^165^
				Progression of lung adenocarcinoma	Binds with 3′‐UTR of YAP mRNA, increasing its stability and thus enhancing exosome secretion, tumour malignancy and drug resistance.	^199^
				Progression of HNSCC	Enhances mitochondrial activity and intracellular energy metabolism, ensuring continuous energy supplies.	^200^
				Progression of BCa	Binds and stabilises PKM2 mRNA, enhancing PKM2‐mediated glycolysis.	^201^
				Progression of BC	Binds with the NEAT1 lncRNA promoter region, enhancing its transcription.	^118^, ^150^
				Suppression of colon adenocarcinoma		^105^
				Inhibition of adipogenesis	Recognises and exports YBX2 and CDKN1A mRNAs into the cytoplasm, leading to increased YBX2 and CDKNIA protein expression levels which inhibit adipogenesis.	^202^
				Promotion of myogenesis	Recognises and exports SMO mRNA into the cytoplasm, leading to increased SMO protein expression levels which promote myogenesis.	^203^
				Promotion of retrovirus replication		^204^
				Promotion of abdominal aortic aneurysm and infiltration of CD45^+^ leukocytes and CD3^+^ T cells		^205^
	YBX1	mRNA	m5C‐modified mRNAs	Progression of GC	Recognises and binds with NSUN2‐mediated m5C sites on FOXC2 mRNA to stabilise it.	^188^
				Progression of BCa	Stabilises oncogenic HDGF mRNA by targeting the m5C‐modified site on its 3′‐UTR and recruiting ELAVL1.	^23^
				Progression of glioblastoma de		^206^
				Progression of CRC		^207^
				Progression of cholangiocarcinoma	Recognises and stabilises m5C‐modified NKILA.	^162^
				Suppresses the development of ccRCC	YBX1/ELAVL1 complex binds and stabilises PEBR1 mRNA, which negatively modulates ccRCC.	^208^
				Progression of PCa	Recognises and binds with NSUN2‐mediated m5C sites on AR mRNA to stabilise it.	^147^
				Progression of epithelial ovarian cancer	Modulates the expression of a variety of downstream targets, including CD44, thus enhancing chemoresistance.	^168^
				Progression of cervical cancer	Recognises and binds with NSUN2‐mediated m5C sites on KRT13 mRNA to stabilise it.	^167^
				Embryonic brain development		^206^
				Maternal‐to‐zygotic transition	Recognises and stabilises m5C‐modified mRNAs by recruiting Pabpc1a, preventing maternal mRNA decay.	^123^

Abbreviations: 5‐hmC, 5‐hydroxymethylcytosine; AR, androgen receptor; BC, breast cancer; BCa, bladder cancer; ccRCC, clear cell renal cell carcinoma; CRC, colorectal cancer; eRNA, enhancer RNA; ESCC, esophageal squamous cell carcinoma; GC, gastric cancer; HCC, hepatocellular carcinoma; HNSCC, head and neck squamous cell carcinoma; HPSCC, hypopharyngeal squamous cell carcinoma; lncRNA, long non‐coding RNA; NPC, nasopharyngeal carcinoma; PCa, prostate cancer; TME, tumour microenvironment; TNBC, triple‐negative breast cancer.

Barriers persist in advancing research on RNA m5C modification, with the ultimate objective of comprehensively understanding the modification and facilitating clinical translation. In the realm of RNA m6A modification detection and research trends, homogeneous techniques are continually evolving to enhance resolution and sensitivity. However, the limitation and pivotal breakthrough lie in RNA methylation detection, where traditional high‐throughput methods for m5C site identification prove to be inefficient. Advancing this field will require integration of computational and experimental approaches, ensuring high sensitivity and specificity in detecting and mapping m5C modifications. These efforts will lay the foundation for applying m5C research to clinical diagnostics and therapeutic interventions.

## AUTHOR CONTRIBUTIONS

Linhui Zhang and Yuelong Li wrote the original manuscript. Liqing Li and Fei Yao reviewed and revised the manuscript. Maoping Cai, Dingwei Ye and Yuanyuan Qu designed, reviewed, edited and funded acquisition. All authors contributed to the writing and revision of the manuscript, and approved its submission.

## CONFLICT OF INTEREST STATEMENT

The authors declare they have no conflicts of interest.

## ETHICS STATEMENT

The authors have nothing to report.

## Data Availability

No data are associated in the manuscript.
